# The Construction and Meaning of Race Within Hypertension Guidelines: A Systematic Scoping Review

**DOI:** 10.1007/s11606-024-08874-9

**Published:** 2024-07-01

**Authors:** Anna Awolope, Hannah El-Sabrout, Anurima Chattopadhyay, Stephen Richmond, Danielle Hessler-Jones, Monica Hahn, Laura Gottlieb, Na’amah Razon

**Affiliations:** 1 School of Medicine, University of California, Davis (UC Davis), Sacramento, CA USA; 2 School of Medicine, University of California, San Francisco (UCSF), San Francisco, CA USA; 3 School of Public Health, Joint Medical Program, University of California, Berkeley, CA USA; 4 Amherst College, Amherst, MA USA; 5 Primary Care and Population Health, Stanford University, Stanford, CA USA; 6 Department of Family and Community Medicine, UCSF, San Francisco, CA USA; 7 Department of Family and Community Medicine and Social Interventions Research and Evaluation Network (SIREN), UCSF, San Francisco, CA USA; 8 Department of Family & Community Medicine, UC Davis, Sacramento, CA USA

**Keywords:** hypertension, health equity, guidelines, race, racism

## Abstract

**Background:**

Professional society guidelines are evidence-based recommendations intended to promote standardized care and improve health outcomes. Amid increased recognition of the role racism plays in shaping inequitable healthcare delivery, many researchers and practitioners have critiqued existing guidelines, particularly those that include race-based recommendations. Critiques highlight how racism influences the evidence that guidelines are based on and its interpretation. However, few have used a systematic methodology to examine race-based recommendations. This review examines hypertension guidelines, a condition affecting nearly half of all adults in the United States (US), to understand how guidelines reference and develop recommendations related to race.

**Methods:**

A systematic scoping review of all professional guidelines on the management of essential hypertension published between 1977 and 2022 to examine the use and meaning of race categories.

**Results:**

Of the 37 guidelines that met the inclusion criteria, we identified a total of 990 mentions of race categories. Black and African/African American were the predominant race categories referred to in guidelines (*n* = 409). Guideline authors used race in five key domains: describing the prevalence or etiology of hypertension; characterizing prior hypertension studies; describing hypertension interventions; social risk and social determinants of health; the complexity of race. Guideline authors largely used race categories as biological rather than social constructions. None of the guidelines discussed racism and the role it plays in perpetuating hypertension inequities.

**Discussion:**

Hypertension guidelines largely refer to race as a distinct and natural category rather than confront the longstanding history of racism within and beyond the medical system. Normalizing race as a biological rather than social construct fails to address racism as a key determinant driving inequities in cardiovascular health. These changes are necessary to produce meaningful structural solutions that advance equity in hypertension education, research, and care delivery.

**Supplementary Information:**

The online version contains supplementary material available at 10.1007/s11606-024-08874-9.

## INTRODUCTION

Clinical practice guidelines are formulated from expert review of existing evidence to assist providers in the diagnosis and management of disease. Such guidelines have historically emerged from the work of trusted bodies, such as government agencies or professional medical associations, and are assumed to integrate benefit versus harm analysis into recommendations. Scholars and activists alike have brought attention to the pervasiveness of racism across the healthcare sector, including in research and clinical practice, which together raise important questions about how the healthcare sector perpetuates racial inequities in health outcomes.^1–5^ Racism is “an organized social system in which the dominant racial group, based on the ideology of inferiority, categorizes and ranks people into social groups called ‘races’ and uses its power to devalue, disempower, and differentially allocate valued societal resources and opportunities to groups defined as inferior.”^6,7^ As a social system, racism works along multiple layers: structural, cultural, interpersonal, and individual.

A key underexplored question is whether race-based guidelines might serve as a mechanism to mediate harm. For example, scholars criticized the CKD Epidemiology Collaboration’s (CKD-EPI) race-adjusted equations for estimated glomerular filtration rate (eGFR) for the role they played in delaying referrals to specialist care and transplantation for African American and Black patients.^8^ Thus, as of 2021, the National Kidney Foundation and American Society of Nephrology recommend using the CKD-EPI equation without race adjustment.^9,10^

While scrutiny is being applied to several race-based equations (e.g., vaginal birth after delivery (VBAC), spirometry, Atherosclerotic Cardiovascular Disease (ASCVD) risk calculator),^11–15^ race continues to appear in a range of medical standards that do not involve equations or calculations. Similar to race-based equations, race-based algorithms are purported to derive from evidentiary findings and intended to function as decision rules.^16^ However, like calculations rooted in race, race-based algorithms can lead healthcare providers into faulty thinking regarding the implications and efficacy of treatments based on race.^17^

Hypertension affects 47% of adults in the United States (US). Despite decades of Joint National Committee (JNC) guidelines and public health campaigns, hypertension remains a leading cause of stroke, heart, and kidney disease. Across the hypertension control cascade,^18,19^ stark inequities exist: hypertension disproportionately impacts people of color, individuals of low socioeconomic status, and uninsured populations, contributing to higher cardiovascular morbidity and mortality in these communities. Hypertension therefore provides a useful case study for assessing the way guidelines utilize race in treatment algorithms.^20^ To better understand how hypertension guidelines have conceptualized and applied race, we conducted a systematic scoping review of adult US hypertension guidelines.

## METHODS

### Guideline Identification and Selection

**Figure 1:**
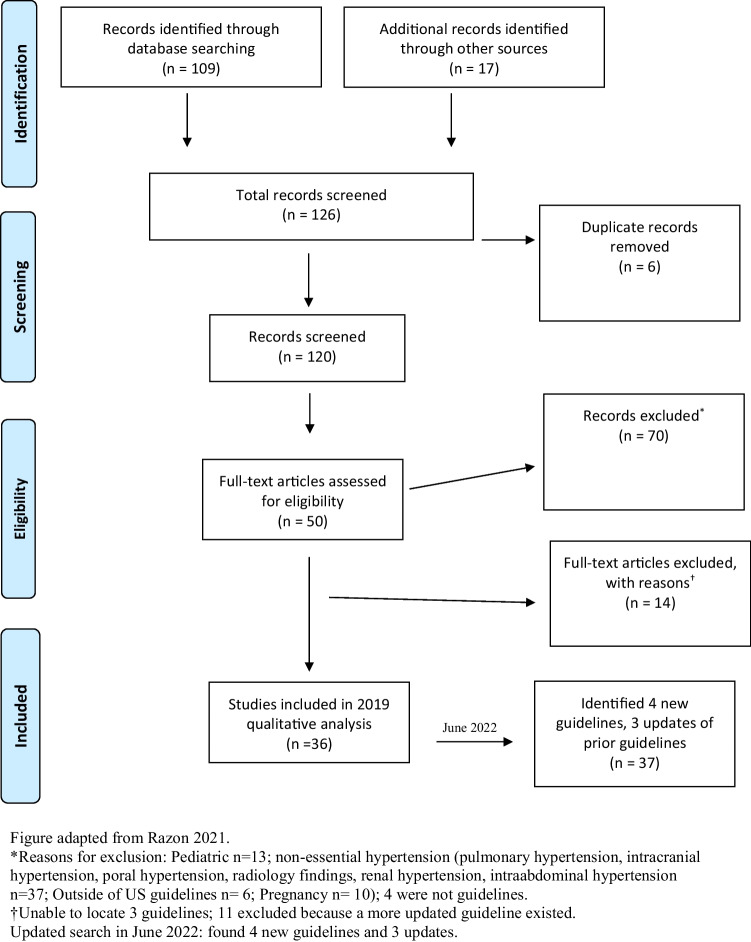
PRISMA inclusion flow diagram.

This paper builds on a previously published systematic scoping review identified 36 hypertension management guidelines published in English in the US between 1977 and December 2019.^21^ Our team included these 36 guidelines and repeated the search utilizing PubMed and American Heart Association databases to identify additional guidelines published between December 2019 and June 2022 using the following search terms: *hypertension guidelines*, *clinical guidelines*, and *clinical recommendations*. After excluding duplicate or updated articles, 37 guidelines were included in the final analysis of this study (Fig. 1 and Appendix A). The final guidelines analyzed were published between 1991 through 2021. Most (62%) were published after 2010. We included only the most updated guideline version (same title and professional society group) with one exception: we included both JNC7 and JNC8 given the difference in scope of the two guidelines.

### Codebook Development and Guideline Assessment

We developed a racial and ethnic category codebook using Ovid, MEDLINE, and Medical Subject Headings (MeSH) terms. Additional terms were added as coding of the guidelines progressed to yield a final list of 23 codes (Table 1).^1^ All 37 guidelines were then coded using Atlas.ti software.^22^

**Table 1:** Race and Ethnicity Categories

Africa/n
Alaska
Arab/Middle Eastern and North African (MENA)
Asia/n
Black
Caucasian
Ethnic
Hawaiian
Hispanic
Indian
Indigenous
Latin
Mexican
Minority/minorities
Native
Other*
Pacific Island
Puerto Rican
Race
Racial
Racism
White

^*^Other included Australian (1); Caribbean (2); Central America (1); Chinese (7); European (5); Filipino (1); Israeli (1); Japanese (4); Korean (1); Mexico (1); South America (2)

Following the initial race category coding, we developed a second codebook drawing on existing literature in the field and an inductive content analysis approach.^2^ Through an iterative process, we identified salient themes of how authors used race categories to yield ten final themes (see Appendix B for detailed definitions). All 37 guidelines were coded with the theme codebook in Atlas.ti, and the frequency of each thematic domain referenced was recorded following the Preferred Reporting Items for Systematic Reviews and Meta-Analysis (PRISMA) (Appendix C). The team initially coded the same four guidelines to reach a consensus on codebook use. The remaining guidelines were then divided among team members. Any questions or disagreements were addressed through continual discussion to reach consensus.

## RESULTS^2^

**Figure 2:**
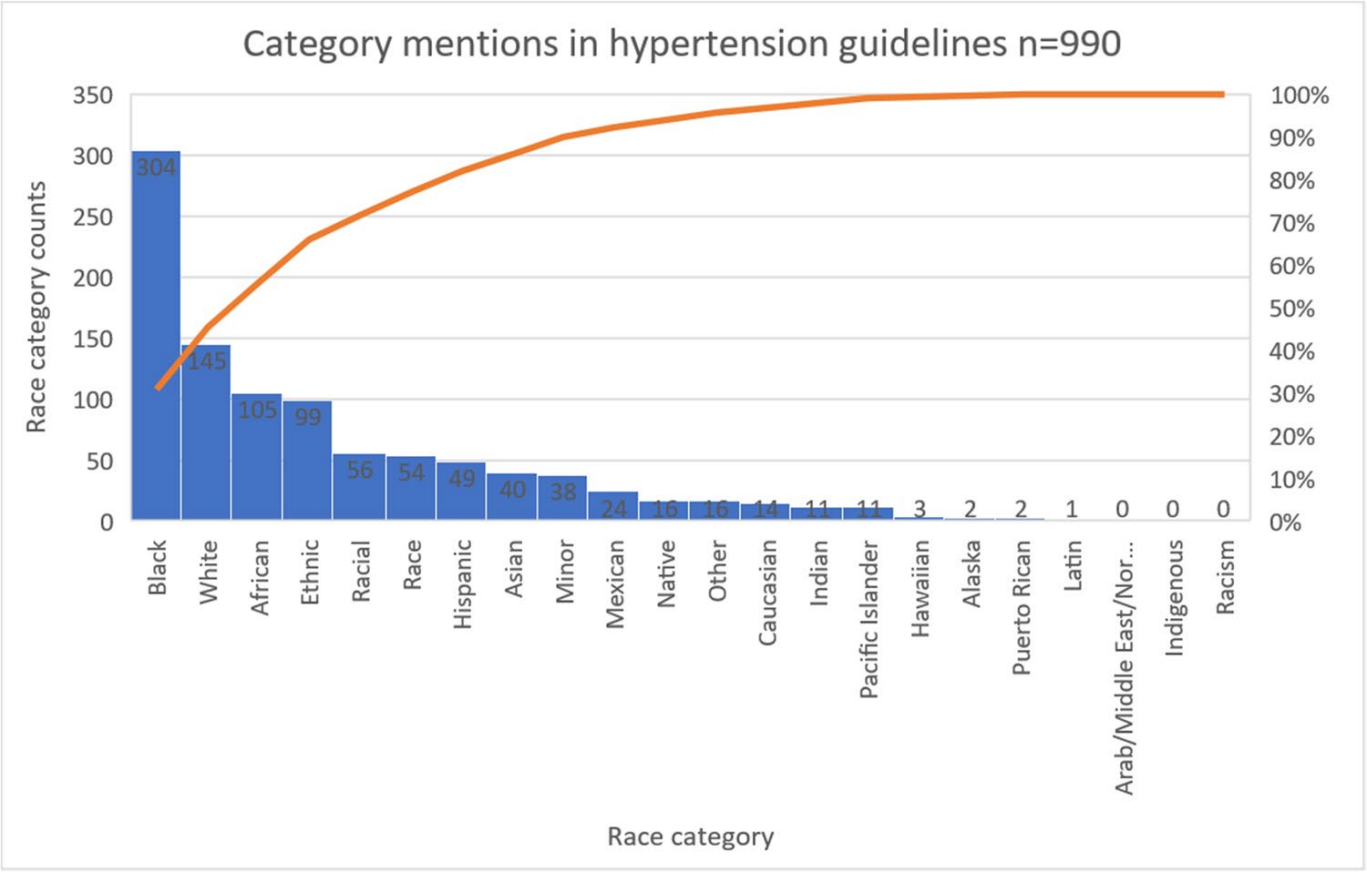
Race category mentions in hypertension guidelines.

Across the 37 guidelines reviewed, 32 referred to racial categories. Within these guidelines, race categories were mentioned 990 times (Fig. 2). The majority of references were to the Black race category (*n* = 304; 31%). White and African were mentioned at 16% (*n* = 145) and 11% (*n* = 105), respectively. Hispanic and Asian categories accounted for 5% (*n* = 49) and 4% (*n* = 40). Other categories, including Caucasian, Indian, Latin, Mexican, Native American, Alaskan, Hawaiian, Pacific Islander, Puerto Rican, and Other^3^were less than 2%. Indigenous and Arab/Middle Eastern/North African categories were not mentioned. The ethnic category accounted for 10% (*n* = 99) of references. Race, racial, and minority accounted for 5% (*n* = 54), 6% (*n* = 56), and 4% (*n* = 38) respectively. We found no mention of the term racism in any guidelines.

### Use of Racial Categories in Hypertension Guidelines

Only two guidelines referenced how race was assigned; both described self-identification.^23,24^ Egan et al. reported, “Data on self-identified non-Hispanic black and white adults,” and Bozkurt et al. referred to “self-described black patients” in discussing pharmacologic treatment decisions. Some guidelines conflated race categories with nationality, ethnicity, or ancestral background. Buzkort et al. merged a discussion of race with national origin, ethnicity, and race categories: “The strength of this association varied among ethnic groups, with the homeostasis model assessment explaining 85.7% of the association in whites, 95.7% in Mexican Americans, and only 32.7% in blacks.”^23^ A 2018 guideline listed particular groups in an epidemiological discussion: “blacks have a higher prevalence of hypertension than that of Hispanic Americans, whites, Native Americans, and other subgroups defined by race and ethnicity.”^25^ However, other guidelines grouped races without any specification, such as in Appel et al.: “On average, blacks have higher BP than nonblacks as well as an increased risk of BP-related complications, particularly stroke and kidney failure.”^26^ Guidelines assumed that individuals with hypertension are categorizable by race and only fit into one category. No guideline discussed how to address individuals who identify with multiple groups or who do not identify with described race categories.

### Meaning of Racial Categories in Hypertension Guidelines

Guideline authors primarily used race in five key domains: describing the prevalence and/or etiology of hypertension; characterizing prior hypertension studies; describing hypertension interventions. While less common, some guidelines described relationships between race and social risk (e.g., financial insecurity)^5,25,27–35^ and others acknowledged the complexity of race categories (Table 2).^25,29,30^

**Table 2:** Thematic Code Count and Examples

Theme	Number of codes	Example
epidemiologic description	202	“According to data from the 1976–1980 National Health and Nutrition Examination Survey II, the combined prevalence of these types of hypertension (systolic blood pressure of 140 mm Hg or higher or diastolic blood pressure of 90 mm Hg or higher or both) is estimated to be 64% in persons from 65 to 74 years of age, with a higher prevalence in blacks (76%) than in whites (63%).”^50^ “Epidemiological data suggest that older age, male gender, and non-Caucasian race are risk factors for faster loss of kidney function in CKD (see K/DOQI CKD Guideline 9), and that older age, male gender, and Caucasian race are risk factors for CVD.”^26^ “Mexican Americans and Native Americans have lower control rates than non-Hispanic Whites and African Americans.”^24^
Pharmacologic treatment	158	“Racial differences in the incidence of antihypertensive drug side effects may occur; African Americans and Asians have a three- to fourfold higher risk of angioedema and have more cough attributed to ACEIs than Caucasians.”^27^ “In INVEST, elderly Hispanic patients with CAD had better BP responses to combination therapy with either a CA plus ACEI or beta blocker plus HCTZ versus white patients.”^21^ “In general, angiotensin-converting enzyme inhibitors are more effective as monotherapy in reducing blood pressure in white patients than in black patients, possibly because the renin-angiotensin system is often less active in black patients.”^35^
Lifestyle, behavior, cultural	79	“The rapid increase in the population of ethnic minorities in the United States is another factor that will lead to a rise in the prevalence of obesity and its complications unless effective, culturally diverse, population-based health promotion strategies are encouraged.”^27^ “Culturally sensitive educational programs and services are needed to educate Hispanics about the importance of taking medication and making lifestyle changes to control their hypertension.”^52^ “Reduced sodium intake and DASH diet should be advocated for prevention and treatment of hypertension, especially in blacks, and response to reduced sodium strengthens with increasing age.”^21^ “Limited awareness (< 30%) and infrequent health care (> 30% 0–1 health-care visits per year) occurred in untreated black and white hypertensive patients without DM/CKD and BP ≥ 140/ < 90 mm Hg.”^25^
Study design	45	“Data on self-identified non-Hispanic black and white adults aged 60 to 79 years in the National Health and Nutrition Examination Survey (NHANES) 2005–2012 were examined as described.”^25^ “This information was organized into a table and reviewed by the ACCF Task Force on Clinical Expert Consensus Documents for writing committee balance across a series of elements including relationships with industry and other entities, regional distribution, sex, race, and specialty area.”^21^
Screening tools and/or laboratory interpretation	37	“In a number of laboratories, serum creatinine is being replaced as an index of renal function by eGFR, the values of which are derived from newer algorithms that include adjustments for gender, race, and age.”^27^ “When BP control was defined as < 140/ < 90 mm Hg for patients with diabetes and/or CKD and < 150/ < 90 mm Hg in adults without diabetes and/or CKD, whites were more likely than blacks to meet target values.”^25^ “In general, the ACC/AHA race- and sex-specific PCE (ASCVD Risk Estimator) should be used for screening and management of hypertension.”^33^
Lack of research	35	“Continued research to examine temporal trends and disparities (with respect to sex, race/ethnicity, and socioeconomic status) in the achievement of performance and quality measures is critical for future revisions of these measure sets. Before adoption of behavioral and motivational strategies as new performance measures, prospective studies evaluating their efficacy in achieving a healthy lifestyle and a standardized process for patient-centered shared decision making for BP control are needed.”^33^ “Unfortunately, sufficient numbers of Mexican Americans, other Hispanic Americans, Native Americans, or Asian/Pacific Islanders have not been included in most of the major clinical trials to allow reaching strong conclusions about their responses to individual anti- hypertensive therapies.”^27^
Social risk/social determinants of health	34	“Future HBP patient registries should include a broader range of races/ethnicities and incorporate data on other socioeconomic determinants of health, as well as patient engagement and activation, to better understand the impact of these variables on medication adherence and BP control.”^33^ “Differential cardiovascular outcomes persist by important sociodemographic characteristics, including but not limited to age, gender, and race/ethnicity. Failure to address the impact of SDoH impedes efficacy of proven prevention recommendations.”^33^
Name of study	11	“Note that the description of the African-American Study of Kidney Disease (AASK) trial is summarized in Wright et al.”^40^ “For example, the Honolulu-Asia Aging Study showed that midlife systolic BP was a significant predictor of reduced cognitive function in later life.”^41^
Genetic	14	“In 3 trials, genetic variation of the angiotensinogen gene modified the BP response to changes in salt intake in nonblacks and the BP responses to weight loss and the DASH diet.”^24^ “Svetkey et al. used an established inpatient protocol to examine the change in BP between intravenous sodium loading and furosemide-induced volume depletion in black US families and found evidence of heritability, although effects of variable family sizes contributed to variation in estimates.”^31^
The complexity of race	20	“This difference, in part, is because Hispanics are not a homogeneous group in terms of genetics, sociodemographics, and health-related lifestyles.”^21^ “Hispanics from Mexico and Central America have lower CVD rates than US whites, whereas those of Caribbean origin have higher rates. Thus, pooling of data for Hispanics may not accurately reflect risk in a given patient.”^23^

*Abbreviations* (in order of appearance): *CKD* chronic kidney disease, *K/DOQI* Kidney Disease Outcomes Quality Initiative, *ACEI* angiotensin-converting enzyme inhibitor, *INVEST* INternational VErapamil SR/Trandolapril Study, *CAD* coronary artery disease, *CA* carbonic antagonist, *HCTZ* hydrochlorothiazide, *DASH* Dietary Approaches to Stop Hypertension, *DM* diabetes mellitus, *BP* blood pressure, *ACCF* American College of Cardiology Foundation, *BMI* body mass index, *ACC* American College of Cardiology, *AHA* American Heart Association, *PCE* pooled cohort equations, *HBP* high blood pressure, *SDoH* social determinants of health

### Prevalence and Etiology of Hypertension

Twenty-three (62%) of the guidelines referred to race categories when describing the prevalence or incidence of hypertension. These included instances when race categories were used as static descriptors alongside epidemiological data. For example, Bozkurt et al. noted, “Hispanics had the highest prevalence of the metabolic syndrome (78.8%) followed by whites (69.5%) and blacks (60.9%).”^23^ In the KDOQI guidelines, authors indicated that “African-American males with hypertension are particularly at risk because they often receive less treatment and, when they do, are less likely to adhere to the treatment regimen.”^27^ In a third of the guidelines,^24,25,27–30,33,36–40^ authors used white as the reference group against which other groups were compared when discussing disease distribution and outcomes.

Eight guidelines (22%) used genetics to explain race differences in hypertension disease. In these cases, authors distinguished racial groups using allele polymorphisms or variations in genetic frequencies. Elijovich et al. concluded that “substantial evidence from different racial and ethnic groups supports a genetic basis for the variation in the BP response to salt.”^41^ Aronow et al. noted: “Japanese appear to have a higher frequency of salt sensitivity than whites, possibly influenced by more prevalent polymorphisms of the angiotensinogen, alpha-adducting, and aldosterone synthase genes.”^30^ Whelton et al. described that “the excess risk of CKD outcomes in at least some blacks with hypertension may be due to the presence of high-risk APOL1 (apolipoprotein L1) genetic variants.”^25^

### Prior Studies

Guidelines referred to race categories and inter-group comparisons used in previously published work. Fifteen guidelines (41%) included at least one description of how a prior study included or excluded racial categories in data collection or analysis. Aronow et al. described the INVEST study as having “compared 8045 Hispanic with 14,531 non-Hispanic hypertensive CAD patients.”^30^ In other cases, an earlier study focused on specific race-defined populations; in several of those cases, the guidelines referenced study titles that included a race category^25,34,42–46^: for example, the African American Study of Kidney Disease (AASK) trial,^25,27,28,30,39,42,44,45^ African-American Heart Failure Trial (A-HeFT),^47,48^ MESA (Multi Ethnic Study of Atherosclerosis) trial,^25^ and REGARDS (Reasons for Geographic and Racial Differences in Stroke).^34^ Sometimes, guidelines highlighted the lack of research on racial groups to draw attention to a knowledge gap for patients defined by race.^24,25,27–30,35,38–40,44,48^

### Hypertension Interventions

Fourteen guidelines (37.8%) relied on race categories to interpret or recommend screening^24,25,32,35,38,44,45,49^ and other clinical measurements.^24,27,32,35,38,45,46,49–52^ At times, race was the sole indicator for a clinical decision, while other guideline authors used race as a component a clinical risk calculator. These included mentions of the use of electrocardiography,^28^ GFR,^27,28,37^ BMI,^29,46^ cardiovascular risk scores (e.g., ASCVD and SCORE),^24,35,46^ salt sensitivity,^33^ and renal artery stenosis.^27^ For example, Egan et al. recommended 135/85 as the “BP [cut-off] for black adults of African descent without DM/CKD.”^24^ Unger et al. asserted that “ethnic-specific cut-offs for BMI and waist circumference should be used.”^46^ Some guidelines suggested race-based laboratory calculations.^27,28,37^

Twenty-two guidelines (59%) mentioned race at least once in reference to pharmacology. Guidelines emphasized the need for different first-line medications based on whether a patient belonged to a “Black” or “African American” racial group. A 1991 guideline asserted that “black patients tend to respond better to diuretics and calcium antagonists than to beta-blockers or angiotensin-converting enzyme inhibitors as monotherapy.”^53^ Whelton et al. recommended “[f]or black adults with hypertension (without HF or CKD), initial antihypertensive treatment should include a thiazide diuretic or CCB.”^25^ The two most recent hypertension guidelines included in this review recommended different pharmacologic medications based on race.^39,46^

Fourteen guidelines (38%) referenced interventions designed to address existing differences in hypertension outcomes between racial groups due to lifestyle or behavioral factors. Ten guidelines discussed interventions to decrease sodium intake^25–28,30,33,34,37,49,53^ and increase potassium intake.^26,30,49^ Multiple guidelines described a unique relationship between salt and high blood pressure in African Americans. From the time this association was mentioned in 1991 (“limiting salt intake is recommended for all hypertensive patients, particularly those who are considered salt-sensitive, such as black and older adults”),^53^ the reference did not substantially change. A 2006 guideline stated: “The potential benefits of these dietary approaches are amplified because survey data indicate that blacks consume high levels of sodium while their potassium intake is less than that of nonblacks.”^26^ The AHA’s 2014 guideline asserted: “A reduction of salt intake is recommended […] in patients who are ‘salt sensitive,’ which may be a fairly common finding in black communities.”^37^ The 2018 guideline on resistant hypertension noted: “Other subgroups of individuals (e.g., those with CKD350 and obesity, blacks) are often more sensitive and can derive particularly robust benefits from sodium restriction.”^34^ Other guidelines highlighted the need for interventions to increase physical activity,^24,25,30,40,49^ decrease obesity rates,^28,29,49,53^ and encourage smoking cessation.^24,49^ For example, Egan et al. wrote, “special attention to smoking cessation is especially important for African Americans with diabetes as they have more lower-extremity amputations than Caucasians with diabetes.”^24^

### Social Risk and Social Determinants of Health

Twelve guidelines (32%) mentioned race when describing social risk factors, e.g., socioeconomic status,^25,27–30,32,35^ insurance status,^27,29,54^ language,^28^ education,^29^ environment/community,^25,27–30,33,34,39^ and transportation/accessibility.^25,27,29–32^ The KDOQI guideline noted: “Nonadherence has been found to be related to lower socioeconomic status, transportation issues, being the child of a single parent, seriousness of the child’s illness, ethnicity, and insurance type.”^27^ Mentions of race in the context of social risk included instances in which race was conflated with socioeconomic status. A 2011 guideline concluded: “Nevertheless, in blacks, socioeconomic status, dietary, and other lifestyle considerations must be examined and addressed, because, to a large extent, these non-drug aspects of elevated BP are of primary significance.”^30^ JNC7 described how “much of the variance in hypertension-related sequelae across racial or ethnic groups may be attributable to differences in socioeconomic conditions.”^28^

### The Complexity of Race

Eight guidelines (22%) made a statement acknowledging the complexity of race and/or that individual populations are not homogenous and thus should not be treated as such in research or clinical care.^23–25,27,29,30,39,46^ Smith et al. highlighted that “Asian Americans and Pacific Islanders often are grouped in the same category; however, not only does the term ‘Asian American’ refer to diverse ethnic subgroups but also Asian Americans in general have different BMI levels than do Pacific Islanders.”^29^ Aronow et al. noted, “Hispanics are not a homogeneous group in terms of genetics, sociodemographics, and health-related lifestyles.”^30^ Several guidelines did acknowledge the experiences of discrimination and the importance of building trust with specific communities. The KDOQI guideline noted: “In providing services or programs to minority populations, it is important to consider the impact that discrimination and prior experience with the health care system may have on health-seeking behaviors and adherence to the prescribed regimen.”^27^ The VA recommended using an “empathetic and non-judgmental approach facilitates discussions sensitive to gender, culture, ethnic, and other considerations.”.^39^

## DISCUSSION

Our scoping review of hypertension guidelines demonstrates race as a frequently cited factor in determining how medical professional societies and practitioners understand, study, and intervene on hypertension in the US. Guidelines from the 30-year period between 1991 and 2022 were reviewed with the majority (62%) containing language on race and racialized categories. The combined categories of Black and African American made up 45% of race category mentions. While Black and African Americans suffer the highest rates of hypertension, other groups also known to experience high rates were seldom mentioned (Hispanic 8%; Asians 4%; Pacific Islander 1%; Native/Indigenous 3%). White, the most prevalent category after Black and African American, made up 16% of mentions and was used in multiple guidelines as the standard of comparison for hypertension rates and treatment, implicitly defining white as the norm. Racism was not explicitly named within the guideline literature, neither as a determinant of high blood pressure nor as an intervention target within the hypertension care cascade.

**Figure 3:**
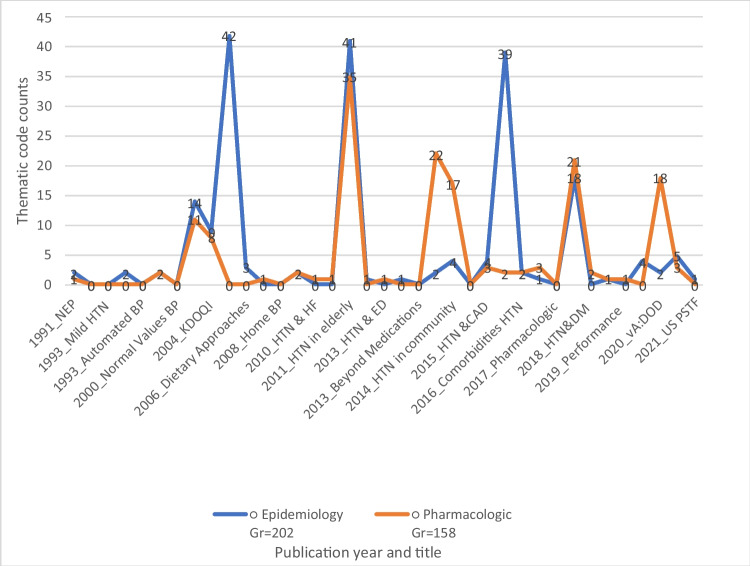
Temporal trend of themes in hypertension guidelines.

While much has changed over the review period pertaining to the management of hypertension, little evolution was seen in the use and meaning of race (Fig. 3 and Appendix D). Hypertension guidelines based on expert analysis of the most current evidence have largely carried forward historical and flawed understandings of race as a biological construct and provided a conduit for translation into clinical practice. While discussions around the causes of inequity in hypertension outcomes have gradually shifted to include social risk, race rather than racism remains centered as an operative factor.

Although race is a social construct, the way in which it is used in hypertension guidelines promotes a biological and fixed interpretation at the level of clinical practice and research.^50,55^ Since race categories were not clearly defined, definitions must be assumed or inferred based on normative, yet unscientific, standards that imbed imprecision into guideline frameworks. These factors lead to uncertainties in practice around applications of guideline algorithms when a patient’s race is unknown or identified as multiracial. For example, while a signal may be found in research indicating that some individuals who identify as Black respond better to a particular treatment protocol, the heterogeneous nature of race means that this protocol may not be best for all individuals who share a Black identity. This monolithic regard for race that situates disparity in biological difference is not only flawed but distracts from important mediators of racism such as access to healthy foods, environments, and healthcare. Thus, we believe that the continued use of race in this way potentiates harm, primarily by implicating race rather than racism as the explanatory (causal) factor of racial disparities in hypertension. Guidelines not only have missed opportunities to use race to better understand the ill effects of racism on health, but also highlight racism as a root cause and describe best practices for eliminating racial disparities in hypertension by targeting racism specifically.

The two guidelines exhibiting the highest frequency of race category mentions were published in 2005 and 2011, respectively. While this may suggest a potential decline in the utilization of race categories in hypertension clinical guidelines, it is important to note that the third and fourth guidelines with the most mentions were published within the last decade, one of which included the highest uses of the “Black” and “white” race categories (Appendices A and D). A similar trend appears when looking at thematic codes, specifically those pertaining to pharmacology and epidemiology. Additionally, the adoption of language related to complexity and social risk was first used in 2004, with the highest frequency occurring in guidelines published in 2005. Although utilization of these codes has expanded across guidelines in recent years, most discussions on the complexity of race categories remain vague.

Our review findings should be interpreted in relation to several limitations. Despite our best efforts, and consultation with a medical librarian, we may have inadvertently excluded a guideline or incorrectly identified a code. Given the consistency of our findings, we do not believe this would significantly change our results. Our team analyzed the content of hypertension guidelines, not the primary studies referenced. Reviewing the use of race categories in the primary literature or its use historically in hypertension guidelines may provide additional information on how race categories are constructed. Nonetheless, the guidelines are a main source consulted by physicians in daily clinical practice. Finally, our review included guidelines over three decades. Earlier guidelines are less likely to be used today and may reflect outdated use of race terms, yet the consistency of the terms and themes suggests that our use of race and lack of attention to racism remain persistent.

## RECOMMENDATIONS

In 2020, the AHA issued a call to action to address structural racism in cardiovascular disease, acknowledging the lack of attention to historical context and the influence of structural racism in its statements on cardiovascular health.^5^ Our review underscores the way in which historical conceptions of race become codified into clinical practice guidelines on hypertension and, like other race-based calculators and decision rules, constitute a form of structural racism. Our review begins to answer that AHA call and broadens its focus of action to include perhaps the most important intersection of cardiovascular research and clinical care—clinical practice guidelines.

**Figure 4:**
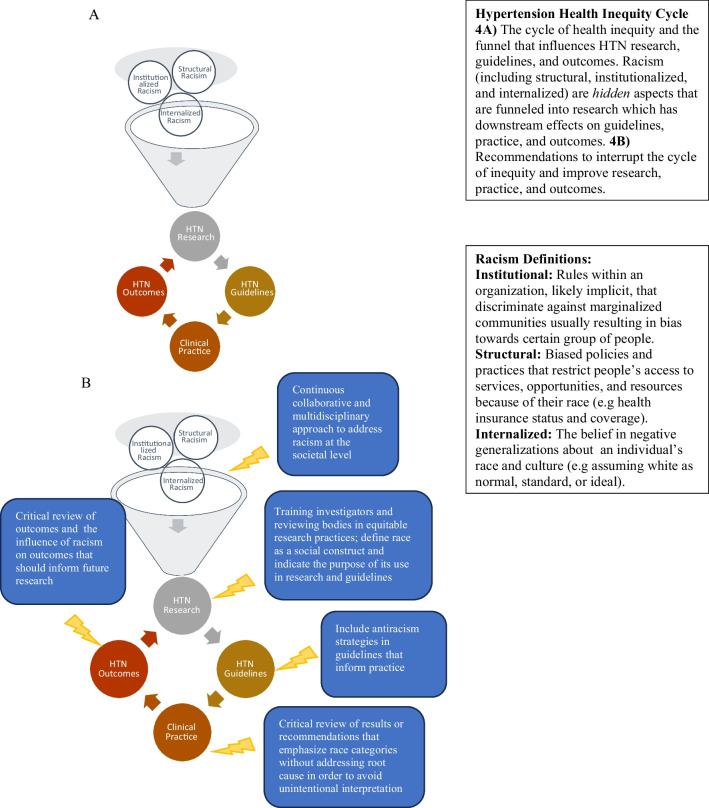
Hypertension health inequity cycle.

Our team developed the Hypertension Health Inequity Cycle (Fig. 4A) to illustrate racism’s influence on the spectrum of hypertension discourse. Institutional, structural, and internalized racism influence this cycle, but are not addressed in hypertension guidelines, nor referenced in the research on which it is based. Improvement in hypertension outcomes must start with addressing racism in the factors that influence research, education, and clinical practice, which are a shared responsibility of investigators, review bodies, professional societies, and clinicians. Figure 4B demonstrates action that should be taken to interrupt this cycle and move towards equitable practice and outcomes.

Based on our appraisal of the use and meaning of race and racialized categories in hypertension guidelines, we provide the following recommendations for mitigating potential harm:

First, guideline authors should always define race and explain its rationale for use.^51^ Definitions should acknowledge race as a social (not biological) construct that mediates the ill health effects of racism, especially within communities of color. This will engender a more critical lens toward research findings, allowing guideline authors to highlight flawed notions of race and model alternative approaches.^52^ Furthermore, funding agencies, researchers, and publishers should rely on standard categories such as those suggested by the Office of Management and Budget.^56^

Second, guideline authors must recognize and name racism as a root cause of racial disparities in hypertension outcomes and describe the role antiracism strategies can play in the hypertension care cascade. Whereas race-based strategies have attempted to correct these disparities by targeting the body and behavior of minoritized individuals, antiracism strategies target the systems that minoritize them. Examples of useful antiracism strategies may include (1) training on interpretation of race-based data or (2) guidance on recognizing mediators of racism that affect patients and best practices for addressing them.

Lastly, guideline authors should collaborate with persons having expertise in racial theory, particularly scholars of color working at the intersection of racial equity and medicine (See Appendix E). This should include looking to existing movements aimed at reexamining race-based practices within specific medical areas, such as eGFR,^50,57–59^ VBAC,^60^ and pediatric urinary tract infections.^50^ These efforts emphasize the necessity of medical assessments based on symptoms and laboratory findings rather than assumptions based on race. This is a necessary step to ensure that guideline-creating bodies not only include diverse racial representation but that guidelines themselves emerge from a deep understanding of race and racism and their impact on health.

## Supplementary Information

Below is the link to the electronic supplementary material.

Supplementary file1 (DOCX 21 KB)

Supplementary file2 (DOCX 13.8 KB)

Supplementary file3 (DOCX 24 KB)

Supplementary file4 (DOCX 84 KB)

Supplementary file5 (DOCX 15 KB)

## Footnotes

1 We use race category to refer to the fixed term used in guidelines but refer in our discussion to these categories as a racialized category to underscore the process that creates and applies these categories to particular people.

2 The inconsistencies, variability, and misuse of race that emerge across hypertension guidelines are a part of the findings of this review. Race is a social construction based on historical and social context. Yet, when researching and writing an article on the use of race categories in medicine, the authors acknowledge the struggle of how to best share the flawed use of categories in the text while also highlighting their problematic use. In the results that follow, we share the language—including exact terms and capitalization—to provide readers with the landscape of race categories in hypertension guidelines. We chose to follow the equity-focused language suggested in the AMA’s *Guide on Language, Narrative and Concepts* which recommends usage of the terms “white” and “Black.”

3 See Table 1 for a list of included terms in the category “Other.”.

## Abbreviations

*AASK*: African American Study of Kidney Disease
*ACC*: American College of Cardiology
*ACCF*: American College of Cardiology Foundation
*ACEI*: Angiotensin-converting enzyme inhibitor
*AHA*: American Heart Association
*A-HeFT*: African American Heart Failure Trial
*APOL1*: Apolipoprotein L1
*ARB*: Angiotensin receptor blocker
*ASCVD*: Atherosclerotic cardiovascular disease
*BB*: Beta blocker
*BMI*: Body mass index
*BP*: Blood pressure
*CA*: Carbonic antagonist
*CAD*: Coronary artery disease
*CKD*: Chronic kidney disease
*DASH*: Dietary Approaches to Stop Hypertension
*DM*: Diabetes mellitus
*eGFR*: Estimated glomerular filtration rate
*HBP*: High blood pressure
*HCT*Z: Hydrochlorothiazide
*HF*: Heart failure
*INVEST*: INternational VErapamil SR/Trandolapril Study
*JHS*: Jackson Heart Study
*JNC*: Joint National Committee
*K/DOQI*: Kidney Disease Outcomes Quality Initiative
*LVH*: Left ventricular hypertrophy
*MENA*: Middle Eastern and North African
*MESA*: Multi-Ethnic Study of Atherosclerosis Trial
*MeSH*: Medical Subject Headings
*PCE*: Pooled cohort equation
*PRISMA*: Preferred Reporting Items for Systematic Reviews and Meta-Analysis
*REGARDS*: Reasons for Geographic and Racial Differences in Stroke
*SCORE*: Systematic COronary Risk Evaluation model
*SDoH*: Social determinants of health
*US*: United States of America
