# Supplementary material for: The Construction and Meaning of Race Within Hypertension Guidelines: A Systematic Scoping Review
Source: J Gen Intern Med. 2024 Jul 1;39(13):2531–42. doi: 10.1007/s11606-024-08874-9 (PMC11436586; doi:10.1007/s11606-024-08874-9)
Supplement: Supplementary file 1 — Supplementary file1 (DOCX 21 KB) [file 11606_2024_8874_MOESM1_ESM.docx]

Appendix A: Hypertension Guidelines Reviewed (Adapted from Razon 2021)

| **Title** | **Author(s)** | **Year** | **Race Categories** | **Themes** |
| --- | --- | --- | --- | --- |
| National Education Program Working Group Report on the Management of Patients with Hypertension and High Blood Cholesterol | Working Group on Management of Patients with Hypertension and High Blood Cholesterol | 1991 | (3) Black  (1) Racial  (1) White | (2) Epidemiologic Description  (1) Lifestyle, Behavior, Cultural  (1) Pharmacologic Treatment |
| Weight Training in Coronary Artery Disease and Hypertension | Stewart | 1992 | no codes | no codes |
| Physical Activity, Physical Fitness, and Hypertension | American College of Sports Medicine | 1993 | (1) Asian  (2) Black  (1) Caucasian  (2) Minority/Minorities  (1) Race  (4) White | (2) Epidemiologic Description  (3) Lack of Research  (1) Lifestyle, Behavior, Cultural  (1) Study Design |
| Ambulatory Blood Pressure Monitoring and Blood Pressure Self-Measurement in the Diagnosis and Management of Hypertension | Appel & Stason | 1993 | no codes | no codes |
| 1993 Guidelines for the management of mild hypertension: Memorandum from a WHO/ISH meeting | Guidelines Sub-Committee of the WHO/ISH (International Society of Hypertension) Mild Hypertension Liaison Committee | 1993 | no codes | no codes |
| Preserving Renal Function in Adults With Hypertension and Diabetes: A Consensus Approach | Bakris et al. | 2000 | (5) African  (1) Hispanic  (1) Mexican  (2) Native | (2) Epidemiologic Description  (2) Name of Study  (2) Pharmacologic Treatment |
| Normal Values of Blood Pressure Self-Measurement in View of the 1999 World Health Organization-International Society of Hypertension Guidelines | Weisser et al. | 2000 | no codes | no codes |
| The Seventh Report of the Joint National Committee on Prevention, Detection, Evaluation, and Treatment of High Blood Pressure | National Heart, Lung, and Blood Institute (United States Department of Health and Human Services) | 2004 | (18) African  (2) Asian  (6) Black  (3) Caucasian  (9) Ethnic  (3) Hispanic  (3) Mexican  (7) Minority/Minorities  (3) Native  (1) Pacific Islander  (5) Race  (7) Racial  (11) White | (14) Epidemiologic Description  (1) Genetic  (2) Lack of Research  (4) Lifestyle, Behavior, Cultural  (2) Screening and/or Lab  (3) Social Risk  (11) Pharmacologic Treatment |
| Goals of Antihypertensive Therapy in CKD | National Kidney Foundation Kidney Disease Outcomes Quality Initiative (NKF KDOQI) | 2004 | (22) African  (1) Black  (7) Caucasian  (5) Ethnic  (4) Hispanic  (2) Indian  (3) Minority/Minorities  (5) Native  (4) Race  (3) White  (1) Other | (1) Complexity / Social Construction  (9) Epidemiologic Description  (3) Lack of Research  (10) Lifestyle, Behavior, Cultural  (5) Screening and/or Lab  (4) Social Risk  (2) Study Design  (8) Pharmacologic Treatment |
| Discovering the Full Spectrum of Cardiovascular Disease Minority Health Summit 2003: Report of the Obesity, Metabolic Syndrome, and Hypertension Writing Group | Smith et al. | 2005 | (2) African  (2) Alaska  (12) Asian  (28) Black  (40) Ethnic  (3) Hawaiian  (7) Hispanic  (8) Indian  (9) Mexican  (20) Minority/Minorities  (10) Pacific Islander  (2) Puerto Rican  (3) Race  (30) Racial  (15) White | (7) Complexity / Social Construction  (42) Epidemiologic Description  (3) Genetic  (12) Lack of Research  (11) Lifestyle, Behavior, Cultural  (1) Screening and/or Lab  (9) Social Risk  (2) Study Design |
| Dietary Approaches to Prevent and Treat Hypertension: A Scientific Statement From the American Heart Association | Appel et al. | 2006 | (14) Black  (1) Racial  (1) White | (3) Epidemiologic Description  (1) Genetic  (12) Lifestyle, Behavior, Cultural |
| ASH Position Paper: Treatment of Hypertension in Patients With Diabetes—An Update | Bakris & Sowers | 2008 | (1) African  (1) Caucasian | (1) Pharmacologic Treatment |
| Call to Action on Use and Reimbursement for Home Blood Pressure Monitoring: Executive Summary: A Joint Scientific Statement From the American Heart Association, American Society of Hypertension, and Preventive Cardiovascular Nurses Association | Pickering et al. | 2008 | (1)  Minority/Minorities | (1) Social Risk |
| ACCF/AHA/ACP 2009 Competence and Training Statement: A Curriculum on Prevention of Cardiovascular Disease: A Report of the American College of Cardiology Foundation/American Heart Association/ American College of Physicians Task Force on Competence and Training (Writing Committee to Develop a Competence and Training Statement on Prevention of Cardiovascular Disease) | Bairey Merz et al. | 2009 | (1) African  (1) Black  (2) Ethnic  (1) Minority/Minorities | (2) Epidemiologic Description  (2) Pharmacologic Treatment |
| Managing Patients with Hypertension and Heart Failure | Heart Failure Society of America | 2010 | (1) African | (1) Study Design  (1) Pharmacologic Treatment |
| ACCF/AHA 2011 Expert Consensus Document on Hypertension in the Elderly: A Report of the American College of Cardiology Foundation Task Force on  Clinical Expert Consensus Documents | Aronow et al. | 2011 | (5) African  (6) Asian  (53) Black  (5) Ethnic  (17) Hispanic  (10) Mexican  (4) Race  (3) Racial  (29) White  (5) Other | (4) Complexity / Social Construction  (41) Epidemiologic Description  (1) Genetic  (2) Lack of Research  (5) Lifestyle, Behavior, Cultural  (6) Social Risk  (3) Study Design  (35) Pharmacologic Treatment |
| Combination Therapy in Hypertension | Gradman et al. | 2011 | (1) Black | (1) Pharmacologic Treatment |
| Blood pressure and treatment of persons with hypertension as it relates to cognitive outcomes including executive function | Gorelick & Nyenhuis | 2012 | (1) Asian | (1) Epidemiologic Description  (1) Name of Study |
| Beyond Medications and Diet: Alternative Approaches to Lowering Blood Pressure: A Scientific Statement From the American Heart Association | Brook et al. | 2013 | (1) Asian  (2) Black | (3) Study Design |
| 2014 Evidence-Based Guideline for the Management of High Blood Pressure in Adults Report From the Panel Members Appointed to the Eighth Joint National Committee (JNC 8) | James et al. | 2013 | (32) Black  (1) Ethnic  (3) Race  (2) Racial | (2) Epidemiologic Description  (2) Lack of Research  (1) Name of Study  (1) Screening and/or Lab  (3) Study Design  (22) Pharmacologic Treatment |
| Clinical Policy: Critical Issues in the Evaluation and Management of Adult Patients in the Emergency Department With Asymptomatic Elevated Blood Pressure | Wolf et al. | 2013 | (5) Black | (1) Social Risk  (4) Study Design  (1) Pharmacologic Treatment |
| An Effective Approach to High Blood Pressure Control: A Science Advisory From the American Heart Association, the American College of Cardiology, and the Centers for Disease Control and Prevention | Go et al. | 2014 | (2) Ethnic  (2) Race | (1) Epidemiologic Description  (1) Screening and/or Lab  (1) Social Risk |
| Clinical Practice Guidelines for the Management of Hypertension in the Community: A Statement by the American Society of Hypertension and the International Society of Hypertension | Weber et al. | 2014 | (4) African  (2) Asian  (17) Black  (4) Ethnic  (1) Race  (2) Racial  (8) White | (4) Epidemiologic Description  (1) Genetic  (2) Lifestyle, Behavior, Cultural  (1) Screening and/or Lab  (17) Pharmacologic Treatment |
| Hypertension in African Americans Aged 60 to 79 Years: Statement From the International Society of Hypertension in Blacks | Egan et al. | 2015 | (14) African  (54) Black  (2) Caucasian  (5) Race  (37) White | (1) Complexity / Social Construction  (39) Epidemiologic Description  (1) Lack of Research  (7) Lifestyle, Behavior, Cultural  (5) Screening and/or Lab  (7) Study Design  (2) Pharmacologic Treatment |
| 2014 Hypertension Guideline: Recommendation for a Change in Goal Systolic Blood Pressure | Handler | 2015 | (2) African  (1) Racial | (1) Name of Study  (1) Screening and/or Lab  (1) Study Design |
| Treatment of Hypertension in Patients With Coronary Artery Disease: A Scientific Statement From the American Heart Association, American College of Cardiology, and American Society of Hypertension | Rosendorff et al. | 2015 | (3) African  (1) Black  (1) Ethnic  (2) Race  (2) White | (4) Epidemiologic Description  (1) Lack of Research  (3) Pharmacologic Treatment |
| Contributory risk and Management of Comorbidities of Hypertension, obesity, Diabetes Mellitus, Hyperlipidemia, and Metabolic Syndrome in Chronic Heart Failure | Bozkurt et al. | 2016 | (3) Black  (1) Ethnic  (1) Hispanic  (1) Mexican  (2) White | (2) Complexity / Social Construction  (2) Epidemiologic Description  (2) Pharmacologic Treatment |
| Salt Sensitivity of Blood Pressure: A Scientific Statement From the American Heart Association | Elijovich et al. | 2016 | (1) African  (2) Asian  (15) Black  (3) Ethnic  (5) Race  (2) Racial  (9) White  (4) Other | (1) Epidemiologic Description  (5) Genetic  (14) Lifestyle, Behavior, Cultural  (6) Screening and/or Lab  (1) Social Risk  (7) Study Design  (3) Pharmacologic Treatment |
| Pharmacologic Treatment of Hypertension in Adults Aged 60 Years or Older to Higher Versus Lower Blood Pressure Targets: A Clinical Practice Guideline From the American College of Physicians and the American Academy of Family Physicians | Qaseem et al. | 2017 | no codes | no codes |
| Resistant Hypertension: Detection, Evaluation, and Management: A Scientific Statement From the American Heart Association | Carey et al. | 2018 | (4) Black  (1) Race  (2) Racial | (1) Epidemiologic Description  (1) Genetic  (1) Lifestyle, Behavior, Cultural  (2) Name of Study  (1) Social Risk  (1) Pharmacologic Treatment |
| Hypertension Management in Diabetes: 2018 Update | Passarella et al. | 2018 | (2) Black | (2) Pharmacologic Treatment |
| 2017 ACC/AHA/AAPA/ABC/ACPM/AGS/APhA/ASH/ASPC/NMA/PCNA Guideline for the Prevention,  Detection, Evaluation, and Management of High  Blood Pressure in Adults: A Report of the American College of Cardiology/American Heart Association Task Force on Clinical Practice Guidelines | Whelton et al. | 2018 | (3) African  (9) Asian  (39) Black  (10) Ethnic  (16) Hispanic  (4) Minority/Minorities  (1) Native  (6) Race  (4) Racial  (17) White  (3) Other | (3) Complexity / Social Construction  (18) Epidemiologic Description  (1) Genetic  (3) Lack of Research  (6) Lifestyle, Behavior, Cultural  (3) Name of Study  (1) Screening and/or Lab  (2) Social Risk  (6) Study Design  (21) Pharmacologic Treatment |
| 2019 AHA/ACC Clinical Performance and Quality  Measures for Adults With High Blood Pressure: A Report of the American College of Cardiology/American Heart Association Task Force on Performance Measures | Casey et al. | 2019 | (6) Black  (6) Ethnic  (8) Race  (1) Racial  (1) White | (2) Lack of Research  (4) Lifestyle, Behavior, Cultural  (6) Screening and/or Lab  (3) Social Risk  (1) Pharmacologic Treatment |
| Measurement of Blood Pressure in Humans: A Scientific Statement From the American Heart Association | Muntner et al. | 2019 | (1) Asian  (8) Black  (2) White  (1) Other | (4) Epidemiologic Description  (1) Lack of Research  (2) Screening and/or Lab  (2) Study Design |
| VA/DoD Clinical Practice Guideline for the Diagnosis and Management of Hypertension in the Primary Care Setting | Department of Veteran Affairs & Department of Defense | 2020 | (22) African  (4) Ethnic  (1) Race  (1) White | (1) Complexity / Social Construction  (2) Epidemiologic Description  (3) Lack of Research  (2) Social Risk  (1) Study Design  (18) Pharmacologic Treatment |
| 2020 International Society of Hypertension Global Hypertension Practice Guidelines | Unger et al. | 2020 | (1) African  (3) Asian  (4) Black  (4) Ethnic  (1) Indian  (1) Latin  (2) Other | (1) Complexity / Social Construction  (5) Epidemiologic Description  (1) Name of Study  (3) Screening and/or Lab  (3) Pharmacologic Treatment |
| Screening for Hypertension in Adults: US Preventive Services Task Force Reaffirmation Recommendation Statement | United States Preventive Services Task Force | 2021 | (3) Black  (2) Ethnic  (3) Race  (2) White | (1) Epidemiologic Description  (1) Lifestyle, Behavior, Cultural  (2) Screening and/or Lab  (2) Study Design |

*Among the 909 race category mentions, there were three instances where there was insufficient information in the text to categorize thematic use (2 in Smith 2005 and 1 in Egan 2015).
