# Supplementary material for: The Construction and Meaning of Race Within Hypertension Guidelines: A Systematic Scoping Review
Source: J Gen Intern Med. 2024 Jul 1;39(13):2531–42. doi: 10.1007/s11606-024-08874-9 (PMC11436586; doi:10.1007/s11606-024-08874-9)
Supplement: Supplementary file 2 — Supplementary file2 (DOCX 13.8 KB) [file 11606_2024_8874_MOESM2_ESM.docx]

Appendix B: Thematic code definitions

| **Theme** | **Definition** |
| --- | --- |
| Epidemiological | The utilization of race as a static descriptor when discussing the epidemiology of hypertension including prevalence, incidence, associations, risk factors  OR the generalization of hypertension distribution among racial groups without any other context |
| Genetic | A statement describing genetic differences in racial groups as the etiology for disease differences |
| Lack of Research | A statement describing the absence of research or a need for additional research, including the study of disparities, specific to racial groups without a call to address structural/systemic contributors |
| Lifestyle, Behavior, Cultural | A statement describing differences in hypertension interventions or outcomes between racial groups due to lifestyle (e.g., diet, exercise, behavior) or culture without mention of structural/systemic contributors to these differences |
| Name of Study | Name of study/ titles including race categories |
| Pharmacologic Treatment | A statement describing a difference in recommendations or outcomes of pharmacological treatment between racial groups |
| The complexity of race | A statement acknowledging race as a social construction or fluid category with historical underpinnings |
| Screening Tools and/or Laboratory Interpretation | A statement describing differences in hypertension screening tools or laboratory interpretation between racial categories OR screening tools with the assumptions that different racial groups hold physiologically differences that should be accounted for differently in these tools/laboratory values. |
| Social Risk/ Social Determinants of Health | A statement describing differences in hypertension recommendations, interventions, or outcomes between racial categories due to differences in social risk (i.e., use of race when describing socioeconomic status, community resources, insurance status, or social determinants of health without discussing historical/structural barriers to care that contribute to these differences). This also includes conflation of race as socioeconomic or social risk |
| Study Design | A statement describing race categories or the exclusion of race categories in study design or data collection |
