# Supplementary material for: The Construction and Meaning of Race Within Hypertension Guidelines: A Systematic Scoping Review
Source: J Gen Intern Med. 2024 Jul 1;39(13):2531–42. doi: 10.1007/s11606-024-08874-9 (PMC11436586; doi:10.1007/s11606-024-08874-9)
Supplement: Supplementary file 4 — Supplementary file4 (DOCX 84 KB) [file 11606_2024_8874_MOESM4_ESM.docx]

Appendix D: Frequency of race category mentions by year of guideline publication
