# Supplementary material for: The Construction and Meaning of Race Within Hypertension Guidelines: A Systematic Scoping Review
Source: J Gen Intern Med. 2024 Jul 1;39(13):2531–42. doi: 10.1007/s11606-024-08874-9 (PMC11436586; doi:10.1007/s11606-024-08874-9)
Supplement: Supplementary file 5 — Supplementary file5 (DOCX 15 KB) [file 11606_2024_8874_MOESM5_ESM.docx]

Appendix E

**Guidelines**

The taste of blood is on the tip of my tongue

as the kin of my blood is on the skin of a ginger root.

The root is sliced and the ginger sinks to the bottom

of a pot filled with boiling water and honey.

My ancestry is not a risk factor for illness, but error

orbiting the iris of hate. The fallacy is a blue pigment

of stiff suits, textbook theories, and the eyes of death

who still see my black curls and brown skin and think

savage, but won’t admit it. Guidelines given by scientists

are sterling silver cuffs, erroneous reasoning

by supposed experts, while social conditions remain ignored…

My race does not determine disease, but centuries of survival.

Who abides by the biology of race preserves the house,

and abides by a holocaust of forced sterilizations,

eugenics, and hidden experiments in a doctor’s office.

Truth is indisputable, as in the existence of me and you.

By Thea Matthews
